# Ventricular Tachycardia Induced by a Wandering Nephroureteral Stent

**DOI:** 10.7759/cureus.37720

**Published:** 2023-04-17

**Authors:** Cristian Valdez, Tony Alarcon, Andrew Kim, Hatim Gemil

**Affiliations:** 1 Internal Medicine, Mountainview Hospital, Las Vegas, USA

**Keywords:** ovarian vein, cardiac arrest, cloacogenic carcinoma, ventricular tachycardia, nephroureteral stent

## Abstract

We present a unique case of a 56-year-old female with complex cloacogenic carcinoma history who experienced intraoperative episodes of ventricular tachycardia and pulselessness of unclear etiology. The etiology was later found to be related to a nephroureteral stent that had perforated the right ureter, entered the right ovarian vein, traversed up the inferior vena cava, and nestled in the right atrium.

## Introduction

Ventricular tachycardia (VT) can be a common finding in medical practice, often caused by both benign and life-threatening situations, with monomorphic ventricular tachycardia being the most common type. It is often triggered by delayed afterdepolarization and presents on ECG with diffuse, regular, and uniformly broad QRS complexes. Monomorphic VT is often benign, unlike polymorphic VT, which presents as prolonged QRS complexes of varying morphology due to multiple ventricular foci. Polymorphic VT is mostly due to pathologic abnormalities in repolarization [1].

Treatment of monomorphic ventricular tachycardia is often accomplished with the use of amiodarone or beta-blockers [2]. Treatment of polymorphic VT requires reversal of the offending agent(s) and possible defibrillation and implantable cardioverter-defibrillator (ICD) placement.

## Case presentation

A 56-year-old female, with a significant past medical history of locally advanced cloacogenic carcinoma with colovaginal fistula status post posterior exenteration with bowel reconstruction (colectomy with small bowel resection and reanastomosis), chemotherapy, and radiation complicated by obstructive uropathy (from radiation-induced ureteral strictures) requiring bilateral ureteral stents, presented for macroscopic hematuria eight years later.

Upon admission, urology was consulted for hematuria of unclear etiology, with hemoglobin of 5.6 requiring transfusion of two units of packed red blood cells. The patient underwent a right ureteroscopy, ureteral dilation, retrograde pyelogram, and right double-J ureteral stent exchange. Intraoperatively, bleeding was immediately noticed in the right ureter, however, due to the clot burden, the source could not be identified. The bladder was irrigated and a 20-French 3-way Foley catheter was placed in the bladder and continuous bladder irrigation (CBI) was initiated. Due to persistent postoperative hematuria, a CT abdomen and pelvis without contrast was ordered (due to an acute kidney injury) demonstrating a potential mass in the distal aspect of the right ureteral stent and a suspected bleed in the right renal collecting system. Additionally, bilateral ureteral stents were seen with large bilateral hydroureteronephrosis. No recent prior imaging was available so chronicity was unclear.

The following day, she underwent a renal angiogram, which demonstrated normal renal vasculature without evidence of significant stenosis, pseudoaneurysm, AV fistula, or active hemorrhage. Despite normal findings, she continued to have hematuria. CBI continued for three days, without resolution of the hematuria.

**Figure 1:**
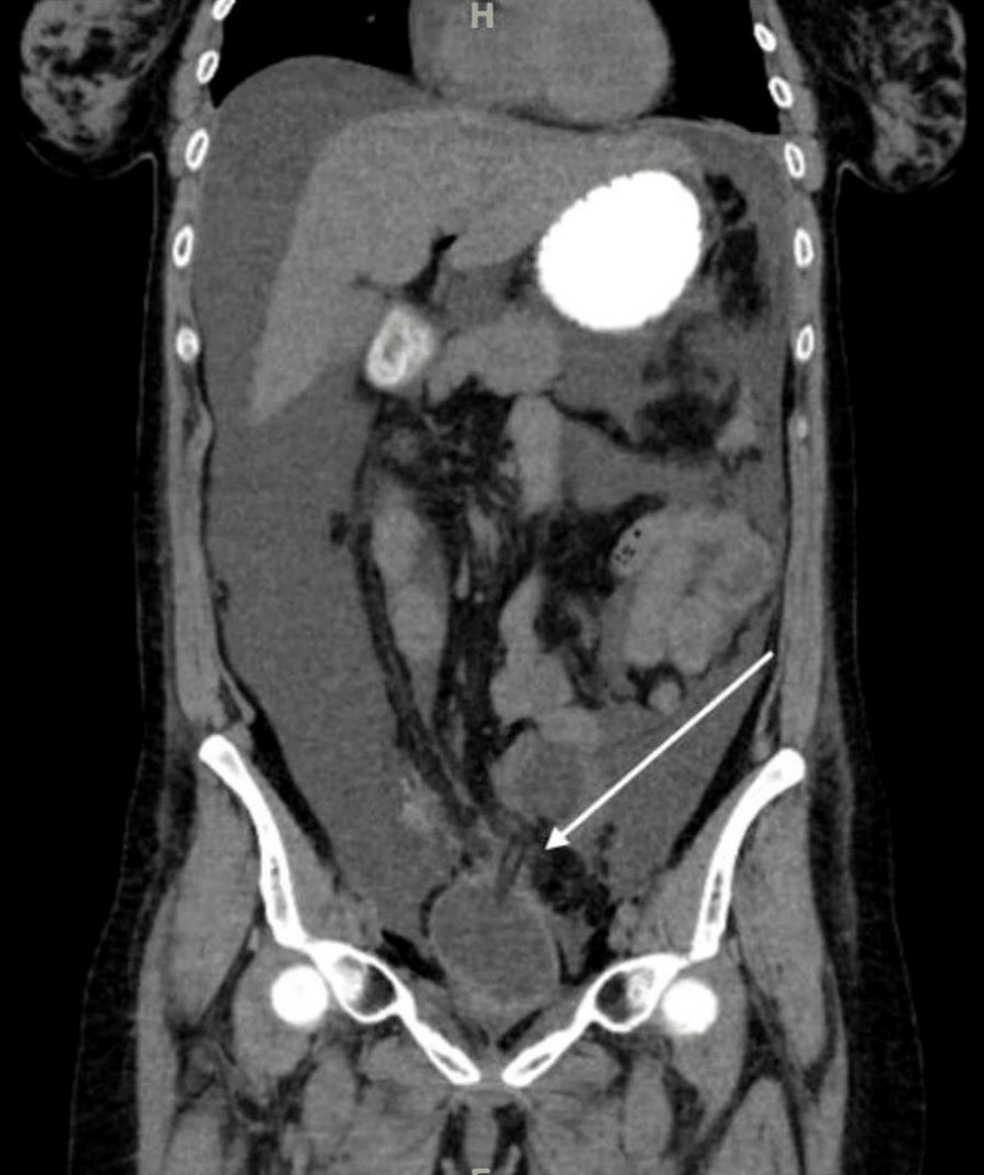
CT abdomen and pelvis without IV or oral contrast The coronal view demonstrates the Foley catheter tip (white arrow tip) extending beyond the urinary bladder dome wall with anterior pneumoperitoneum and perihepatic/colonic ascites.

Due to persistent hematuria, she underwent cystoscopy with right ureteroscopy and right nephroureteral stent exchange. Despite this intervention, her hematuria persisted. Repeat CT abdomen and pelvis without IV contrast (Figure 1) was concerning for a perforated viscus of unclear etiology and worsening bilateral hydronephrosis. Gynecologic oncology and urology opted for conservative management, with continued CBI and close monitoring of hemoglobin.

A rapid response was called the following day for dyspnea, increasing abdominal pain, and distension. The patient’s CBI was noted to be clogged with blood clots. The CBI system was promptly exchanged after manual irrigation failed. Upon exchange, 4 liters of irrigation fluid immediately drained with a resolution of her symptoms. Throughout the rapid response, she remained hemodynamically stable. CT abdomen and pelvis with oral contrast was ordered to further assess for worsening abdominal distension and pain with continued hematuria. Imaging was most notable for bladder wall rupture, interval increase in moderate anterior pneumoperitoneum, and interval increase in moderate ascites (measuring 4 cm in thickness in the perihepatic region, previously 2 cm).

The patient underwent an exploratory laparotomy with the repair of a 1 cm perforation of the bladder, concomitant lysis of adhesions, and release of a distal partial small-bowel obstruction. Mild hematuria persisted after surgery. Four days after the procedure, she was noted to have new episodes of frank blood with clots in the Foley catheter. A repeat CT abdomen and pelvis without contrast was performed demonstrating severe bilateral hydronephrosis.

Due to worsening bilateral hydronephrosis, which was believed to be due to ureteral fibrosis from prior radiation, she required bilateral nephrostomy tubes. During this, a bilateral nephrostogram and antegrade pyelogram were performed for the persistent hematuria, demonstrating a right arterio-ureteral fistula with communication of the distal right ureter with the right iliac artery in the region of the internal/external branch point. This finding prompted a pelvic angiography with embolization of the proximal right internal iliac artery with arterial stent placement from the distal right common iliac to the proximal right internal iliac artery.

**Figure 2:**
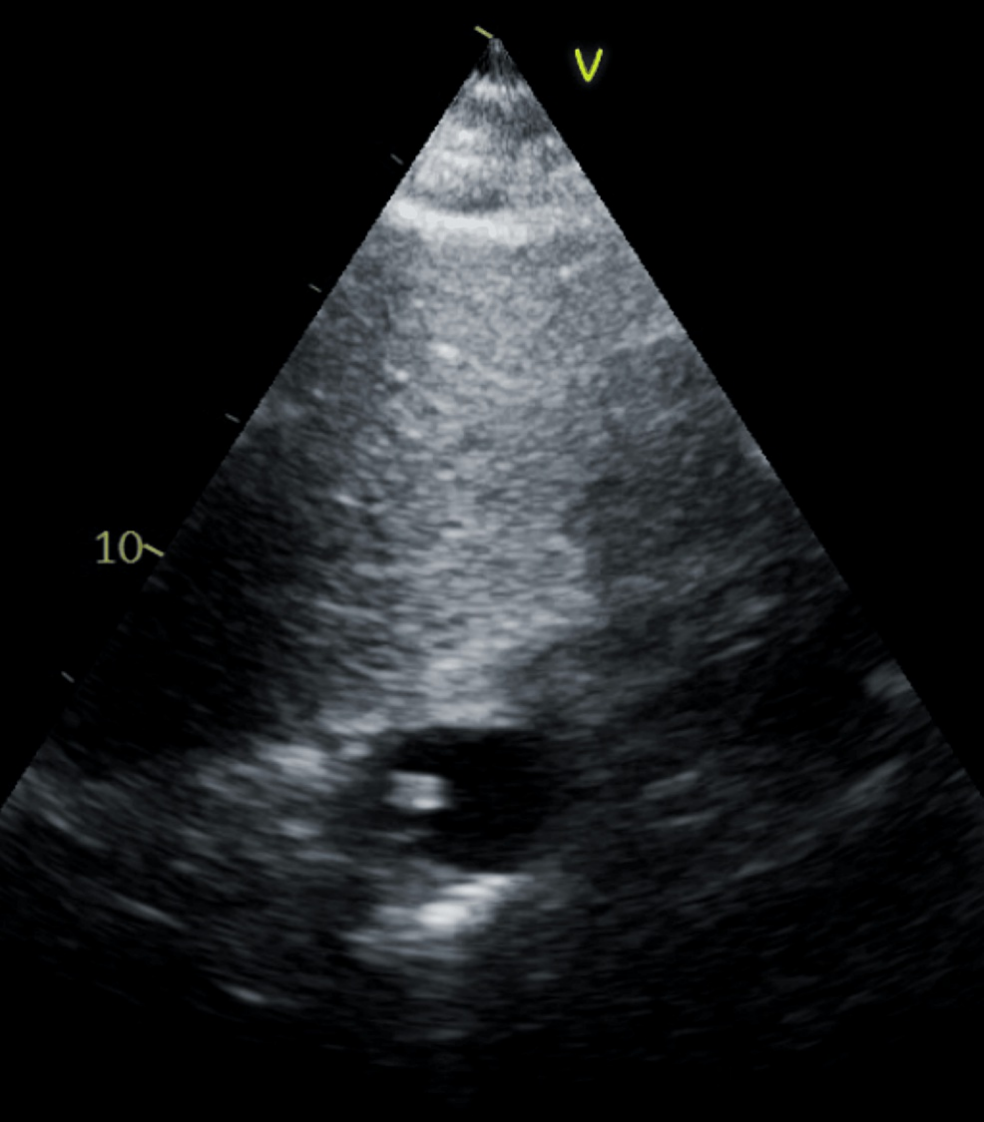
TTE subcostal view Subcostal view of the right atria with an echogenic object TTE: transthoracic echocardiogram

**Figure 3:**
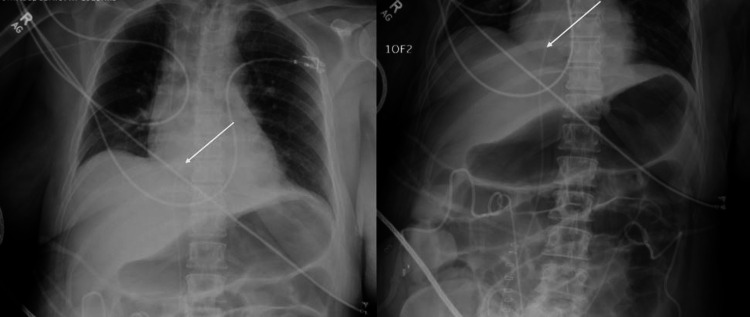
AP chest X-ray (left); KUB (right) AP chest X-ray (left): demonstrating a nephroureteral stent (white arrow) in the IVC extending into the right atrium. This was read as a thin catheter projecting over the right side of the abdomen and in the mediastinum. KUB (right): demonstrating a right nephroureteral stent (white arrow) in the IVC extending into the cardiac silhouette. This was read as bilateral nephrostomy tubes and ureteral stents in situ along with right iliac stent and right internal iliac coils. AP: anteroposterior; KUB: kidney, ureter, and bladder X-ray; IVC: inferior vena cava

Three days after embolization, she underwent an exploratory laparotomy due to refractory hematuria and a 3 g/dL hemoglobin drop. During this, a distal ileal conduit with small bowel resection and re-anastomosis was performed. Intraoperatively, she developed non-sustaining runs of ventricular tachycardia, lasting less than 15 seconds, treated with lidocaine. She then developed a pulseless rhythm for less than 10 seconds before spontaneously converting. She was started on an amiodarone drip with an initial bolus and cardiology was consulted. An echocardiogram showed an ejection fraction (EF) of 60-65%, no regional wall motion abnormalities, mild mitral and tricuspid regurgitation, mild pulmonary hypertension, and an echogenic object seen in the right atrium (Figure 2). She also underwent a rubidium positron emission tomography (PET) myocardial rest and stress scan, which showed no reversible signs of ischemia, along with a chest X-ray and abdominal X-ray (Figure 3). She was discharged on metoprolol and advised to follow up with Cardiology on an outpatient basis for continued workup.

**Figure 4:**
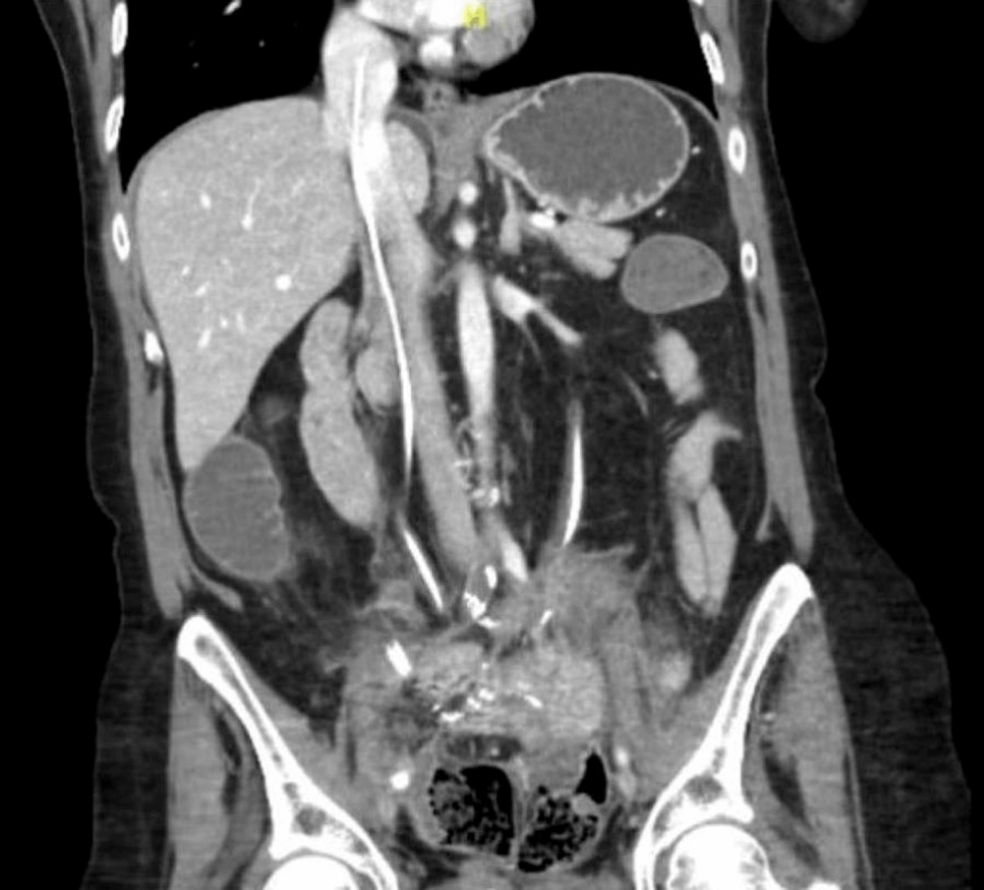
Coronal view CT abdomen and pelvis with IV contrast Demonstrating a right nephroureteral stent in the IVC and right atrium IVC: inferior vena cava

She again presented to the emergency department one week later for abdominal pain and decreased ability to tolerate oral intake. She underwent imaging with CT abdomen and pelvis with IV contrast (Figure 4) and was incidentally found to have a catheter extending from the right atrium inferiorly within the IVC. She was taken to the operating room and found to have a previously failed nephroureteral stent, which perforated the right ureter allowing the catheter to enter the right ovarian vein and traverse up the IVC into the right atrium. The catheter was removed without complication and metoprolol from prior admission was discontinued, as the etiology of her ventricular arrhythmias from prior admission was suspected to be due to the catheter. The patient has had subsequent follow-ups with no further reports of ventricular arrhythmias.

## Discussion

This case highlights multiple learning points, including the importance of personally reviewing imaging and maintaining a broad differential.

Personally reviewing imaging

This case emphasizes the importance of personally reviewing imaging in order to correlate image findings to clinical presentation. Although we could not find any other cases like this in the literature, the overall symptoms and exam combined with history, echocardiogram, and chest X-ray findings, along with persistent hematuria, could have led to a more rapid diagnosis of this patient’s ventricular tachycardia. 

Maintaining a broad differential

Non-sustained ventricular tachycardia (NSVT) is known to occur in up to 3% of healthy males, occurs in 8% of patients with hypertension, and 45-75% of patients with known myocardial infarction (MI) [3,4]. Our patient did not fall under any of these categories, making spontaneous ventricular tachycardia an odd occurrence. Another plausible etiology could be the arrhythmogenic anesthetics the patient received during the procedure, as greater than 10% of patients undergoing general anesthesia have been shown to exhibit abnormal heart rhythms [5]. However, in lieu of her echocardiogram, chest X-ray, surgical history, and her overall symptoms further investigation into non-cardiac-related causes should have been prompted.

## Conclusions

This case serves as a reminder of the importance of daily image reviews for hospitalists and the increased risk of iatrogenic complications in patients with complex surgical and radiation histories. Although the complication presented in this case is extremely rare, it highlights the importance of a thorough chart and patient history review. It acknowledges the delays in treatment and the potential for unnecessary procedures that can result when reviews are inadequate.
